# Supplementary material for: Two New Flavonoids from the Nuts of Areca catechu
Source: Molecules. 2019 Aug 7;24(16):2862. doi: 10.3390/molecules24162862 (PMC6721780; doi:10.3390/molecules24162862)
Supplement: Supplementary file 1 [file molecules-24-02862-s001.pdf]

Supporting Information

Two new flavonoids from the nuts of *Areca catechu*

Mengfei Yuan [†], Yunlin Ao [†], Nan Yao, Jing Xie, Dongmei Zhang, Jian Zhang [*], Xiaoqi Zhang [*] and Wencai Ye

Institute of Traditional Chinese Medicine & Natural Products, and Guangdong Provincial Engineering Research Center for Modernazation of TCM, College of Pharmacy, Jinan University, Guangzhou 510632, P. R. China

[†] They contributed equally to this work.

List of Supporting Information

S3–6. Computational data of **1**.

S7. Cytotoxicity assay

S8. HRESIMS and UV and IR spectra of **1**.

S9–11. 1D and 2D NMR spectra of **1** in CD_3OD.

S12. HRESIMS and UV and IR spectra of **2**.

S13–15. 1D and 2D NMR spectra of **2** in CD_3OD.

Computational data of 1. The systematic random conformational analysis of one pair of enantiomers (2*S*-**1**, 2*R*-**1**) were performed in the SYBYL 8.1 program by using MMFF94s molecular force field, which afforded 12 conformers for **1**, with an energy cutoff of 10 kcal mol^{-1} to the global minima. All of the obtained conformers were further optimized using DFT at the B3LYP/6-31+G(d) level in gas phase by using Gaussian09 software.[1] All of the optimized stable conformers were used for TDDFT computation of the excited stats at the same levels, with the consideration of the first 50 excitations. The overall ECD curves of **1** were weighted by Boltzmann distribution of each conformer (with a half-bandwidth of 0.3 eV). The calculated ECD spectra of **1** were subsequently compared with the experimental ones. The ECD spectra were produced by SpecDis 1.6 software.[2]

Table S1. Key transitions and their related rotatory and oscillator strengths of conformer **1** at the B3LYP/6-31+G(d) level in the gas phase.

**HOMO is 111**					
**No.**	**Energy (cm-1)**	**Wavelength (nm)**	**R (length)**	**Osc. Strength**	**Major contribs**
1	29941.12032	333.9888385	57.0375	0.0439	HOMO->LUMO (96%)
2	32919.7464	303.7690473	-3.5705	0.0109	H-5->LUMO (13%), H-2->LUMO (10%), H-1->LUMO (75%)
3	33660.16848	297.0870454	-27.2091	0.0678	H-3->LUMO (12%), H-2->LUMO (69%), H-1->LUMO (14%)
4	34231.21296	292.1310446	12.6766	0.0036	H-5->LUMO (81%)
5	36383.11504	274.8527714	46.1947	0.2134	H-3->LUMO (78%)
6	36794.46064	271.7800404	0.1195	0.0001	H-4->LUMO (93%)
7	37898.64128	263.8617022	-4.9189	0.0064	HOMO->L+1 (94%)
8	39030.24496	256.2115613	1.0138	0.0087	H-3->L+1 (10%), H-2->L+1 (60%)
9	41033.74	243.7018902	35.284	0.1066	H-3->L+1 (12%), H-1->L+1 (77%)
10	41615.26976	240.2964118	0.6462	0.0007	H-4->L+1 (87%)
11	41856.4312	238.9119118	-17.4058	0.0378	H-3->L+1 (59%), H-2->L+1 (19%), H-1->L+1 (11%)
12	42767.03744	233.8249409	1.7136	0.0016	HOMO->L+2 (72%), HOMO->L+3 (22%)
13	43557.46624	229.5817655	-30.1182	0.0431	H-8->LUMO (25%),

					HOMO->L+2 (15%), HOMO->L+3 (51%)
14	43811.53264	228.2504034	0.6707	0.0016	H-6->LUMO (84%)
15	43929.2904	227.6385507	23.5851	0.0083	H-8->LUMO (40%), H-6->LUMO (13%), HOMO->L+4 (25%)
16	44701.97488	223.7037631	-1.0664	0.0047	H-1->L+2 (52%), H-1->L+3 (23%)
17	44809.24736	223.1682206	-51.8387	0.0833	H-2->L+3 (22%), H-2->L+4 (11%)
18	44902.00176	222.7072203	-21.2865	0.0187	H-2->L+2 (30%), H-2->L+3 (14%), HOMO->L+4 (16%), HOMO->L+5 (17%)
19	45174.61904	221.3632392	23.7348	0.0285	H-2->L+2 (28%), HOMO->L+4 (19%), HOMO->L+5 (23%)
20	45618.22704	219.2106237	-2.2046	0.0032	H-7->LUMO (94%)
21	45983.59872	217.4688428	-63.7337	0.0879	H-1->L+3 (17%), H-1->L+4 (26%), HOMO->L+5 (20%)
22	46311.86864	215.9273701	16.108	0.0322	H-1->L+4 (28%), H-1->L+5 (13%), HOMO->L+6 (11%)
23	46497.37744	215.065893	38.4593	0.0268	H-3->L+2 (29%), H-3->L+3 (10%), HOMO->L+6 (22%)
24	46656.26976	214.3334658	2.4639	0.0008	H-4->L+2 (30%), H-4->L+3 (37%), H-4->L+4 (12%), H-3->L+2 (10%)
25	46741.76512	213.9414285	-13.2479	0.0279	H-3->L+2 (15%), H-2->L+4 (15%), HOMO->L+6 (24%)
26	46899.04432	213.2239611	-13.0012	0.0154	H-2->L+4 (36%), HOMO->L+6 (14%)
27	47178.92064	211.959067	-6.1891	0.0376	H-3->L+4 (14%), H-2->L+5 (13%)
28	47278.93408	211.5106906	-1.324	0.0022	H-5->L+1 (85%)
29	47403.95088	210.9528808	44.5125	0.0271	H-2->L+5 (10%), H-1->L+8 (19%)
30	47601.55808	210.0771572	-16.7411	0.0274	H-3->L+3 (19%), H-3->L+4 (13%), H-2->L+5 (15%), H-1->L+5 (18%)

Table S2. Cartesian coordinates of conformer **1**.

**Standard orientation:**					
**Center Number**	**Atomic Number**	**Atomic Type**	**Coordinates (Angstroms)** X	Y	Z
1	6	0	-4.29183	1.302612	-0.974744
2	6	0	-4.49025	0.023753	-1.50899
3	6	0	-3.93975	-1.08809	-0.88177
4	6	0	-3.18074	-0.92978	0.277546
5	6	0	-2.95955	0.338323	0.804458
6	6	0	-3.52845	1.455712	0.188096
7	6	0	-2.63208	-2.09631	0.979262
8	6	0	-1.92778	-1.83422	2.279217
9	6	0	-1.24523	-0.46698	2.22463
10	8	0	-2.21227	0.565278	1.931069
11	6	0	-0.00933	-0.30293	1.340689
12	6	0	0.575481	-1.36084	0.628381
13	6	0	1.708967	-1.15355	-0.16676
14	6	0	2.306692	0.11557	-0.20417
15	6	0	1.716792	1.198713	0.463485
16	6	0	0.579812	0.973716	1.246451
17	8	0	3.412303	0.31609	-1.00186
18	6	0	4.663929	0.205733	-0.27925
19	6	0	5.446005	-0.99011	-0.84166
20	6	0	5.467459	1.486939	-0.54739
21	8	0	4.955915	2.583584	0.202179
22	8	0	4.922392	-2.22273	-0.35914
23	8	0	-4.17434	-2.32122	-1.42952
24	8	0	-2.81026	-3.21653	0.505777
25	8	0	2.311645	2.423559	0.293137
26	6	0	1.585579	3.563639	0.746828
27	8	0	2.312555	-2.1392	-0.91023
28	6	0	1.489777	-3.24293	-1.28326
29	8	0	-4.89159	2.312212	-1.67794
30	6	0	-4.71799	3.634621	-1.18397
31	1	0	-5.08216	-0.1029	-2.41162
32	1	0	-3.35002	2.430457	0.630141
33	1	0	-1.21179	-2.63613	2.483217
34	1	0	-2.69389	-1.84687	3.063737
35	1	0	-0.89778	-0.26032	3.246132
36	1	0	0.152565	-2.35691	0.697705
37	1	0	0.11961	1.78688	1.802142
38	1	0	4.489105	0.081464	0.798082
39	1	0	5.405824	-1.01473	-1.93585
40	1	0	6.494723	-0.9539	-0.53121

41	1	0	6.514386	1.36609	-0.25296
42	1	0	5.43086	1.763515	-1.60639
43	1	0	3.982457	2.573028	0.083826
44	1	0	3.979175	-2.24494	-0.62527
45	1	0	-3.72727	-2.98735	-0.85101
46	1	0	2.132102	4.455099	0.424267
47	1	0	0.586841	3.605176	0.299379
48	1	0	1.534624	3.582746	1.84009
49	1	0	2.020907	-3.80578	-2.05684
50	1	0	1.334447	-3.91319	-0.43215
51	1	0	0.535569	-2.91109	-1.70673
52	1	0	-5.25237	4.314675	-1.85431
53	1	0	-5.15219	3.742496	-0.18456
54	1	0	-3.66153	3.92257	-1.19022

[1] Gaussian 09, Revision A.02, M. J. Frisch, G. W. Trucks, H. B. Schlegel, G. E. Scuseria, M. A. Robb, J. R. Cheeseman, G. Scalmani, V. Barone, B. Mennucci, G. A. Petersson, H. Nakatsuji, M. Caricato, X. Li, H. P. Hratchian, A. F. Izmaylov, J. Bloino, G. Zheng, J. L. Sonnenberg, M. Hada, M. Ehara, K. Toyota, R. Fukuda, J. Hasegawa, M. Ishida, T. Nakajima, Y. Honda, O. Kitao, H. Nakai, T. Vreven, J. A. Montgomery, Jr., J. E. Peralta, F. Ogliaro, M. Bearpark, J. J. Heyd, E. Brothers, K. N. Kudin, V. N. Staroverov, R. Kobayashi, J. Normand, K. Raghavachari, A. Rendell, J. C. Burant, S. S. Iyengar, J. Tomasi, M. Cossi, N. Rega, J. M. Millam, M. Klene, J. E. Knox, J. B. Cross, V. Bakken, C. Adamo, J. Jaramillo, R. Gomperts, R. E. Stratmann, O. Yazyev, A. J. Austin, R. Cammi, C. Pomelli, J. W. Ochterski, R. L. Martin, K. Morokuma, V. G. Zakrzewski, G. A. Voth, P. Salvador, J. J. Dannenberg, S. Dapprich, A. D. Daniels, O. Farkas, J. B. Foresman, J. V. Ortiz, J. Cioslowski, and D. J. Fox, Gaussian, Inc., Wallingford CT, 2009.

[2] T. Bruhn, A. Schaumlöffel, Y. Hemberger, G. Bringmann, SpecDis version 1.60, University of Wuerzburg, Germany, 2012.

Evaluation of cytotoxicity activities of 1–8

The cytotoxic effects of compounds **1**–**8** on the viability of three human cancer cell lines were determined by the MTT assay. Compounds **1** and **2** exhibited a moderate cytotoxic activity against HepG2 cell lines with IC_{50} values of 49.8 and 53.6 μM, respectively, and the other compounds exhibited no cytotoxicity with IC_{50} values over 100 μM.

Cytotoxicity assay

Human breast cancer MCF-7, hepatocellular carcinoma HepG2, and lung cancer A-549 cell lines were obtained from the American Type Culture Collection (ATCC). The three cells were cultured in the RPMI 1640 medium containing 100 units/ml penicillin and 100 ug/ml streptomycin with 10% FBS in a humidified atmosphere of 95% air and 5% CO_2 (v/v) at 37℃. The three human cancer cell lines were grown in 96-well culture plates for 48 h. After that, the cells were treated with compounds **1**–**8** at various concentrations for 72 h. A 30 μL aliquot of MTT solution (5 mg/mL) was added into each well and incubated for another 4 h. Subsequently, the medium was removed and adding 100 μL of DMSO to dissolve the purple formazan, the absorbance was recorded at 570 nm using a microplate Reader (Thermo scientific multiskan MK3, USA). Each well was performed in triplicate in 3 independent experiments. The concentration giving 50% inhibiton (IC_{50}) was determined from the dose-response curves using Prism software and expressed as the mean ± SD.

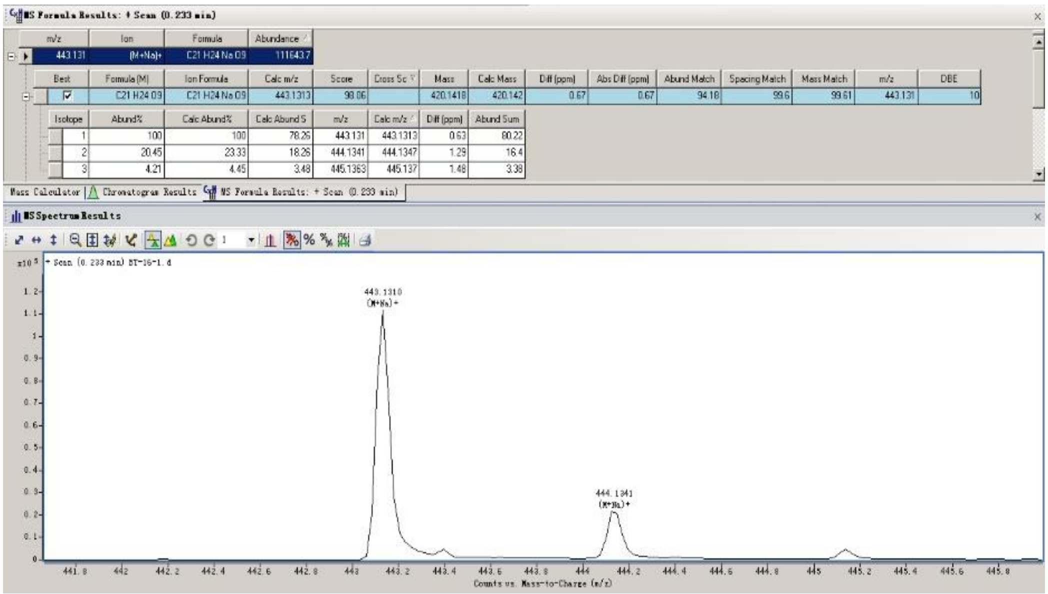

Figure S1. HRESIMS spectrum of **1**

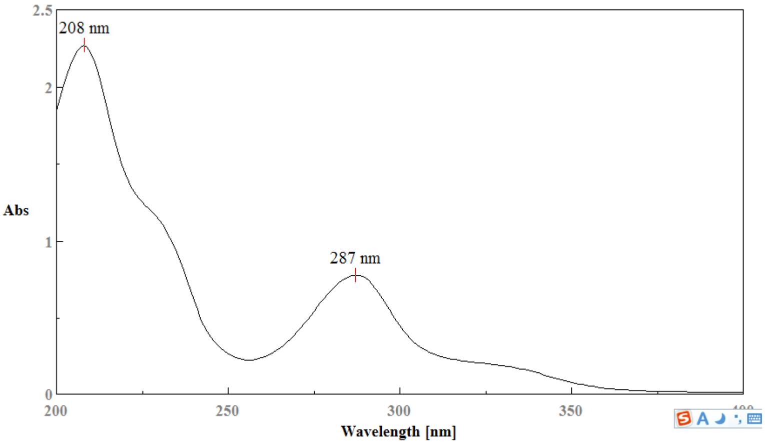

Figure S2. UV spectrum of **1** (MeOH)

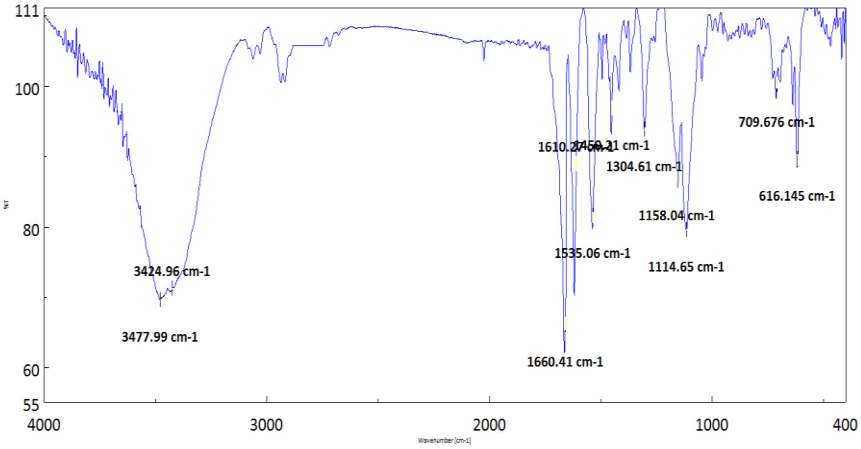

Figure S3. IR spectrum of **1** (KBr disc)

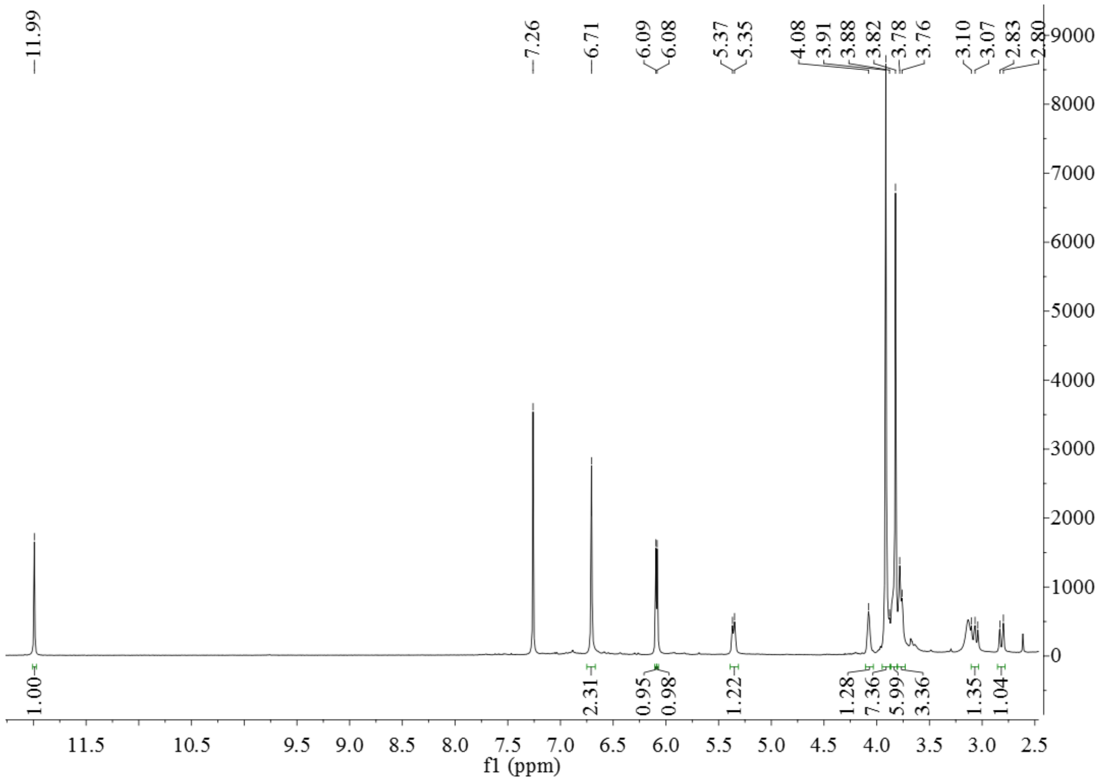

Figure S4. ^{1}H NMR spectrum of **1** (500 Hz, $CDCl_3$)

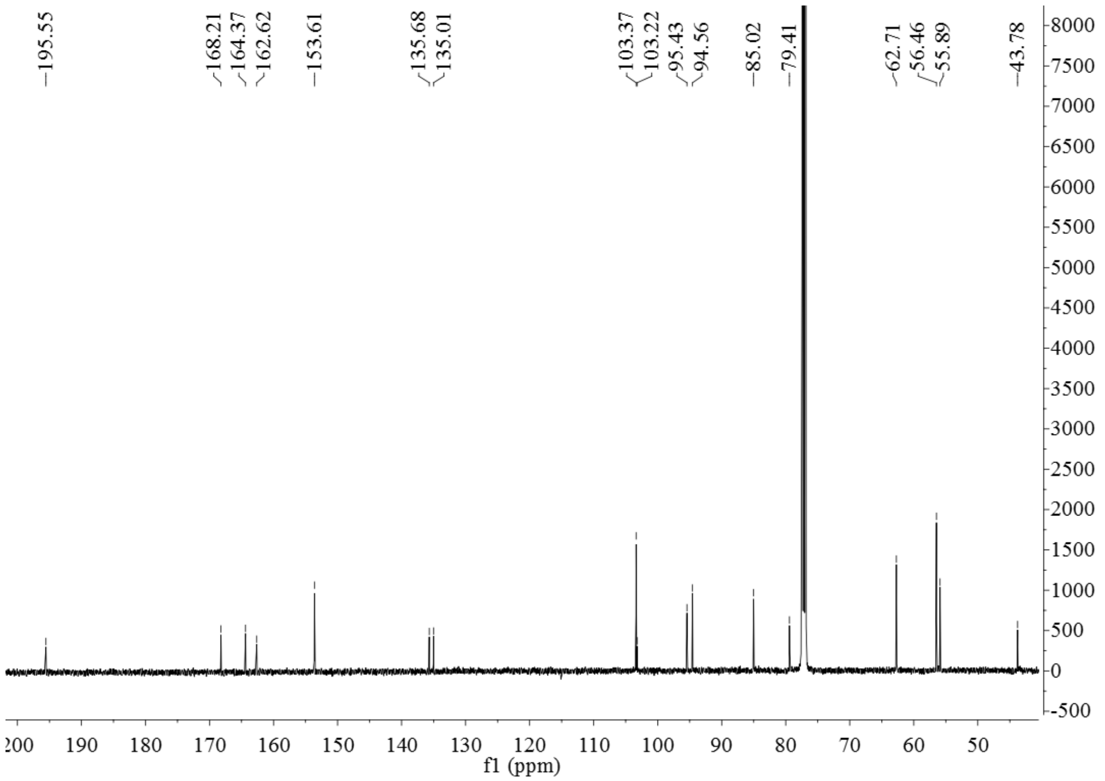

Figure S5. ^{13}C NMR spectrum of **1** (125 Hz, $CDCl_3$)

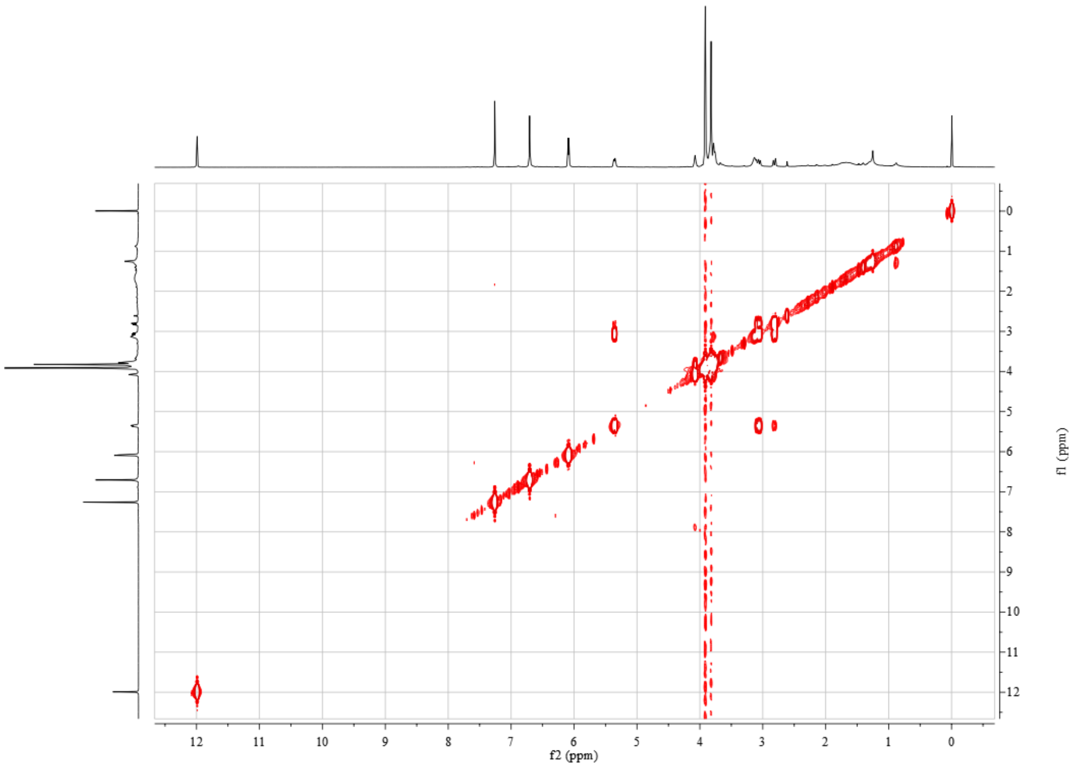

Figure S6. ^{1}H-^{1}H COSY spectrum of **1** ($CDCl_3$)

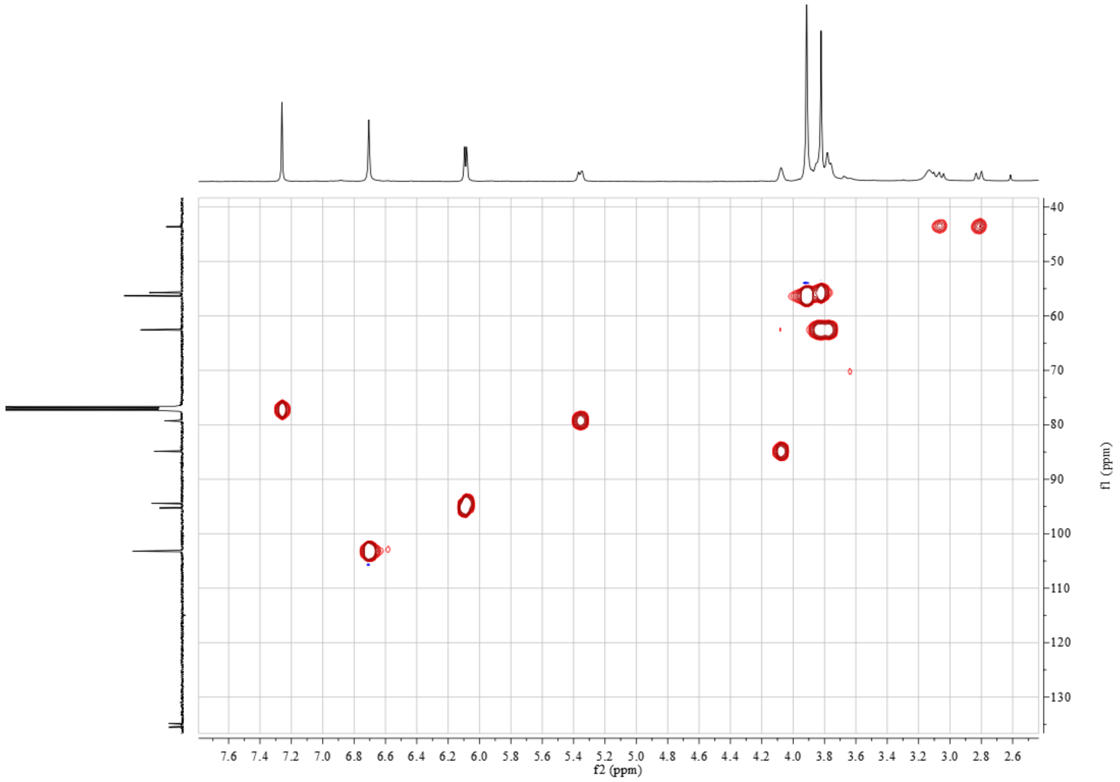

Figure S7. HSQC spectrum of **1** ($CDCl_3$)

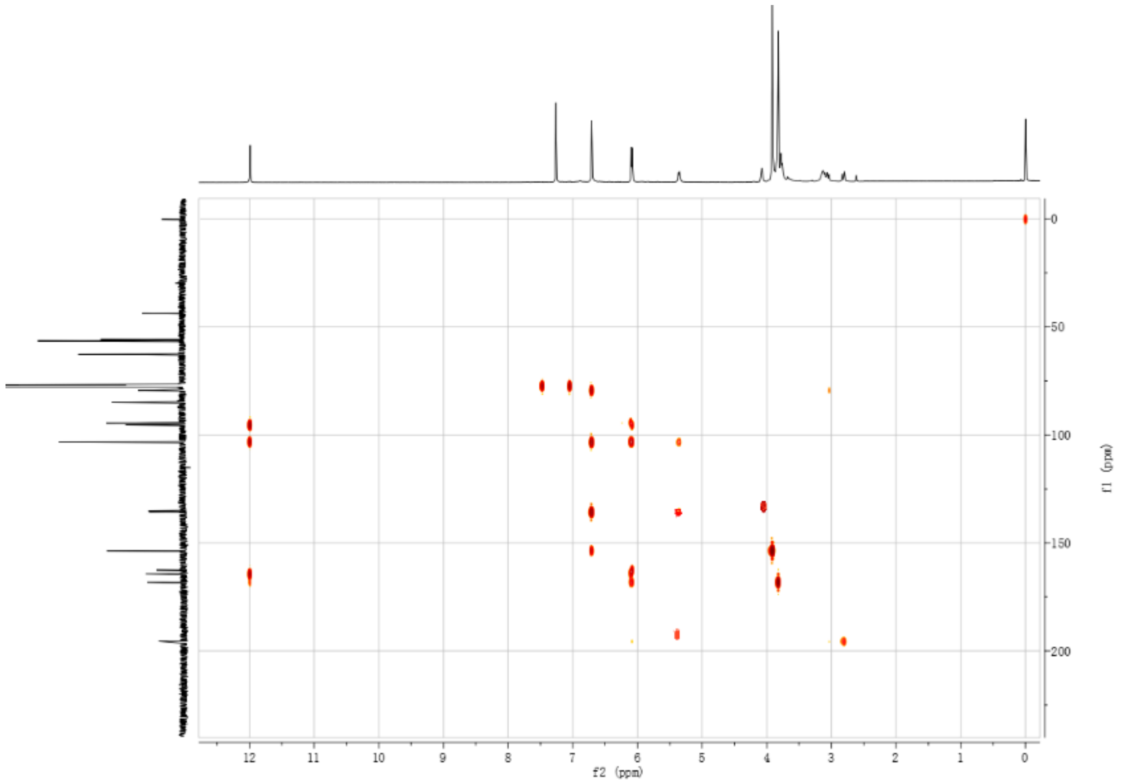

Figure S8. HMBC spectrum of **1** ($CDCl_3$)

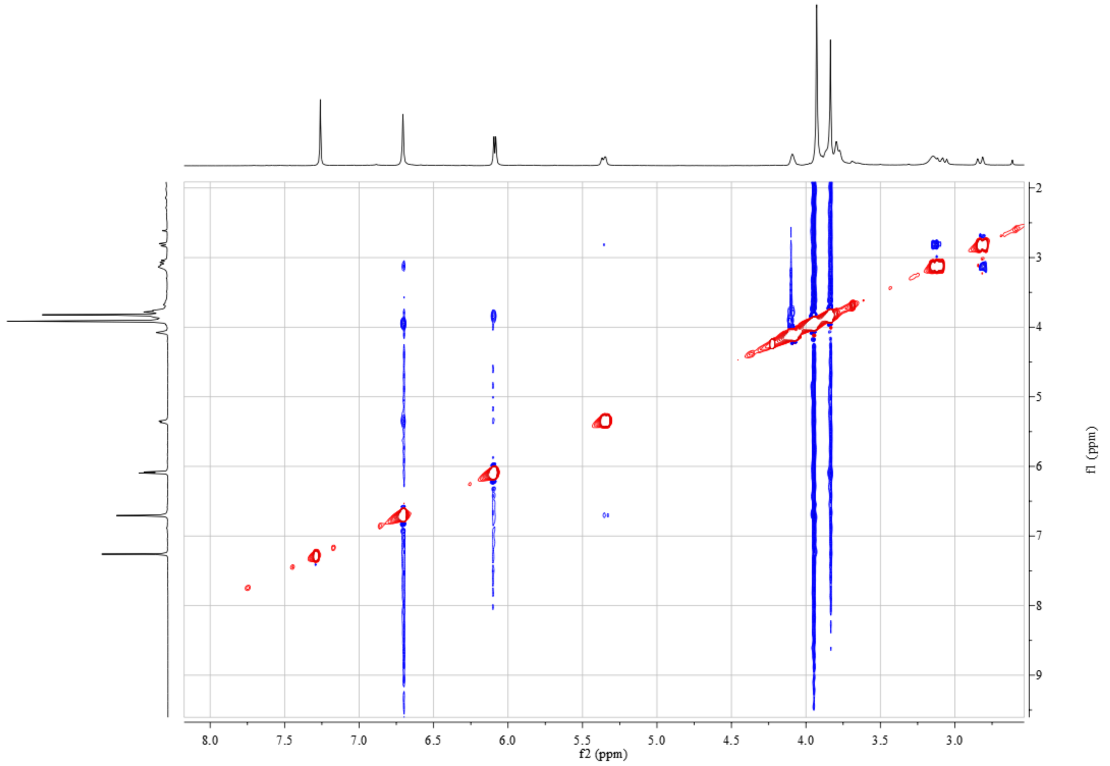

Figure S9. NOESY spectrum of **1** ($CDCl_3$)

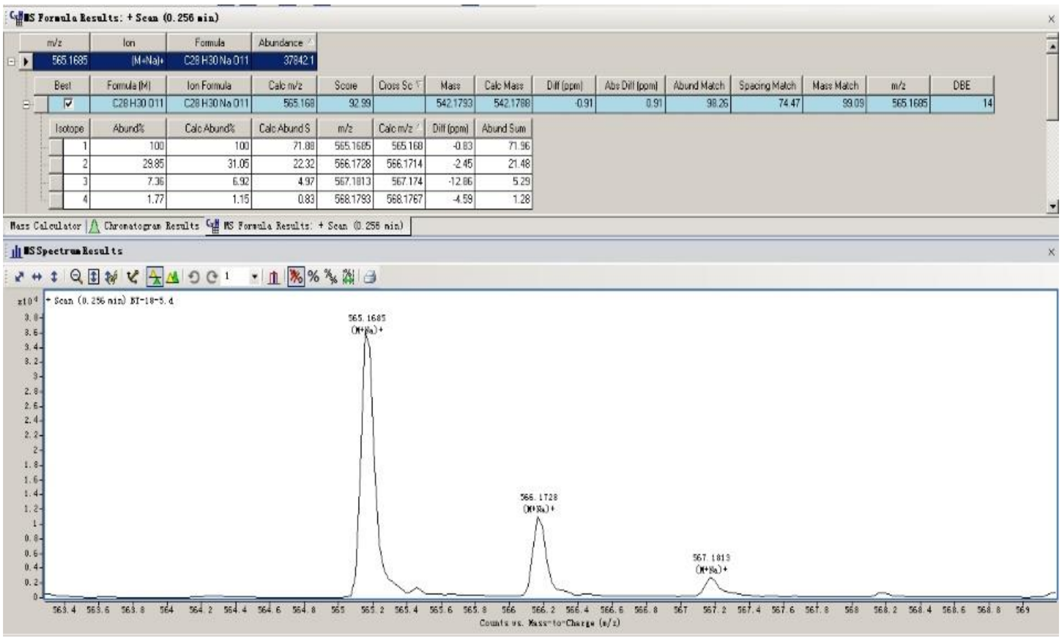

Figure S10. HRESIMS spectrum of **2**

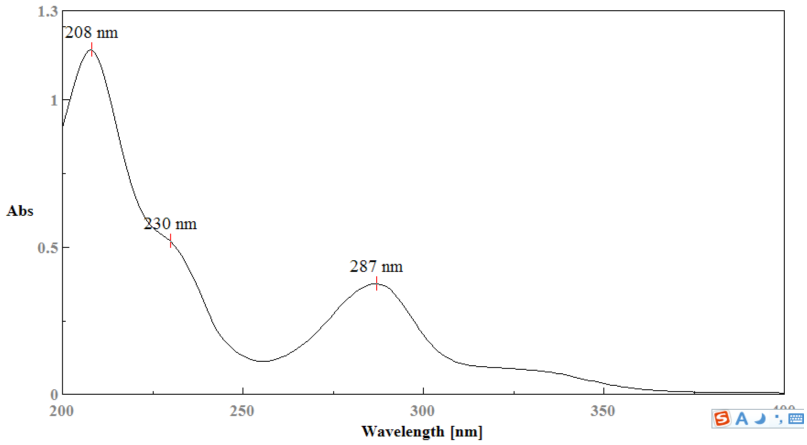

Figure S11. UV spectrum of **2** (CH_3OH)

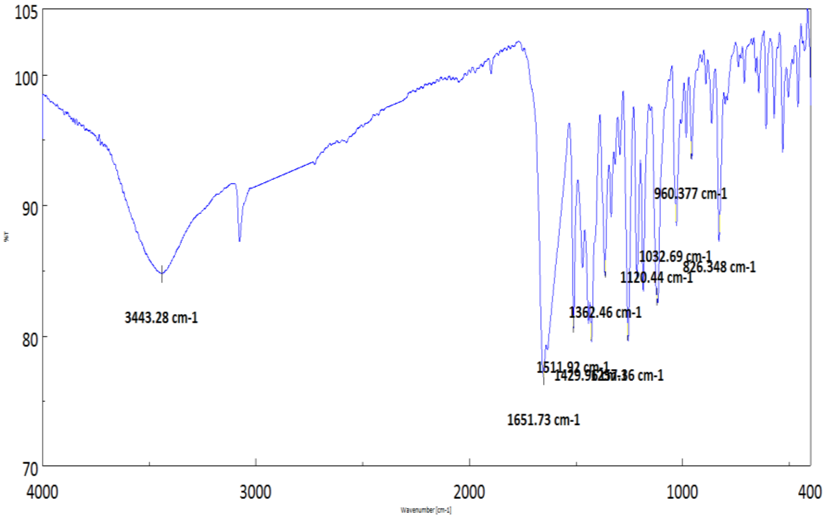

Figure S12. IR spectrum of **2** (KBr disc)

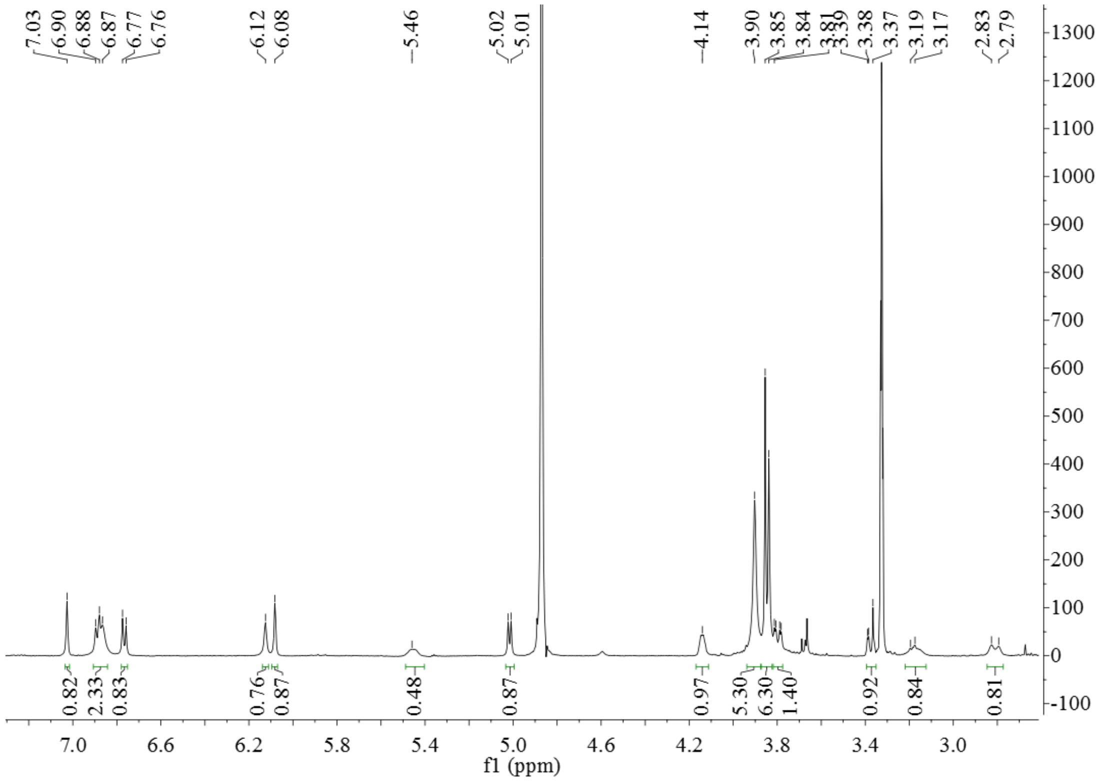

Figure S13. ^{1}H NMR spectrum of **2** (500 Hz, CD_3OD)

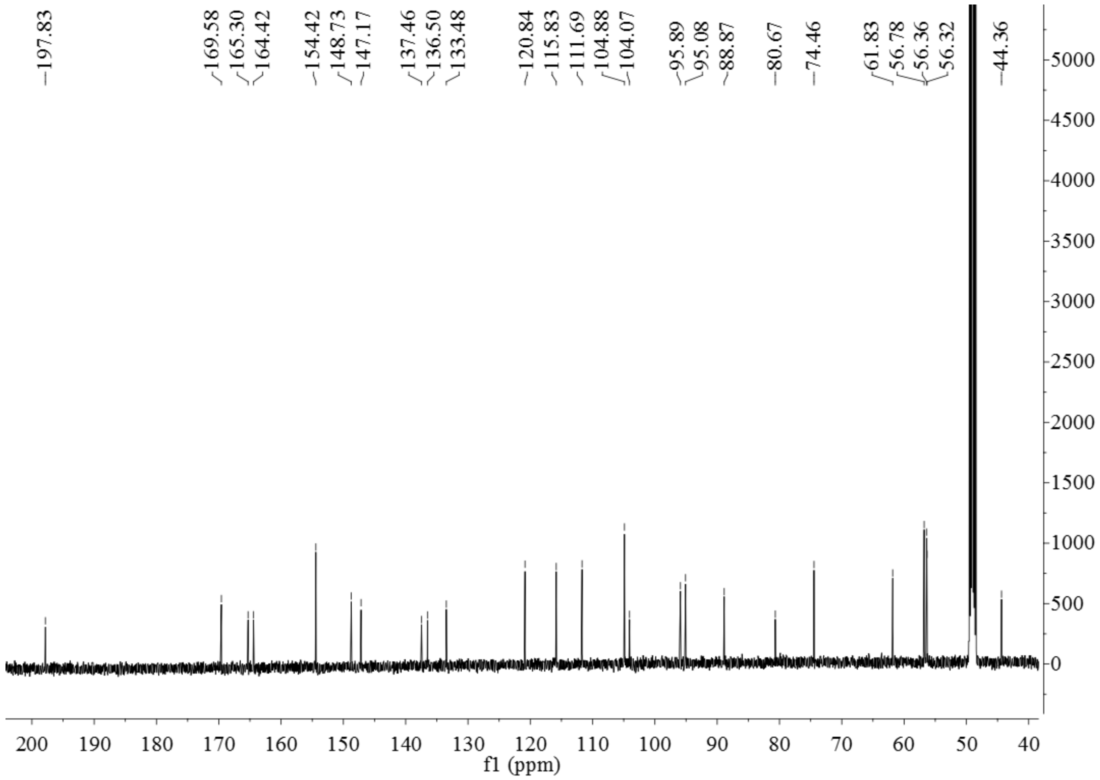

Figure S14. ^{13}C NMR spectrum of **2** (125 Hz, CD_3OD)

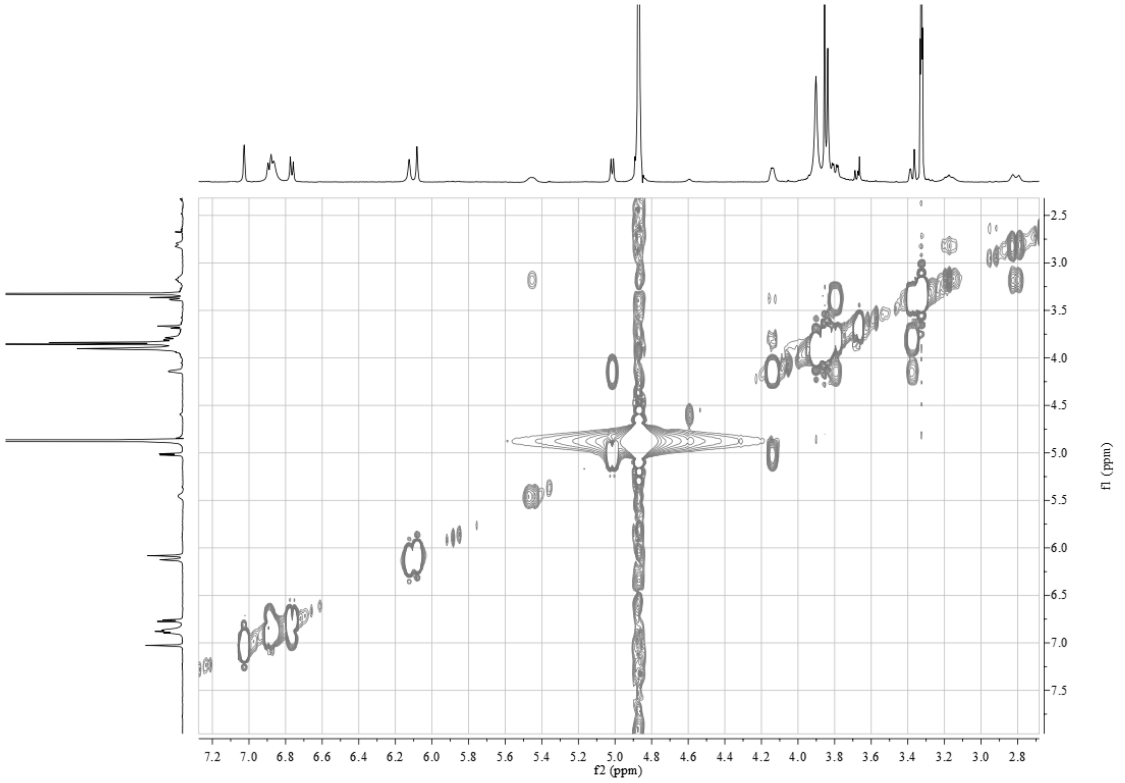

Figure S15. ^{1}H-^{1}H COSY spectrum of **2** (CD_3OD)

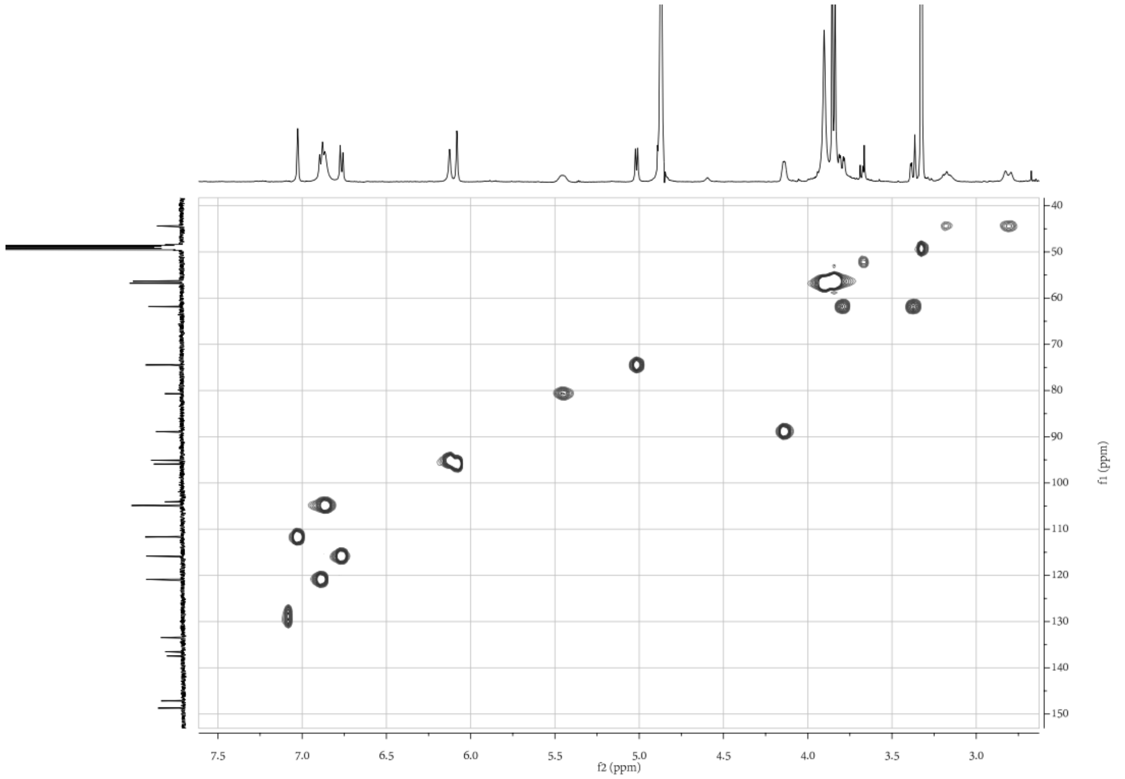

Figure S16. HSQC spectrum of **2** (CD_3OD)

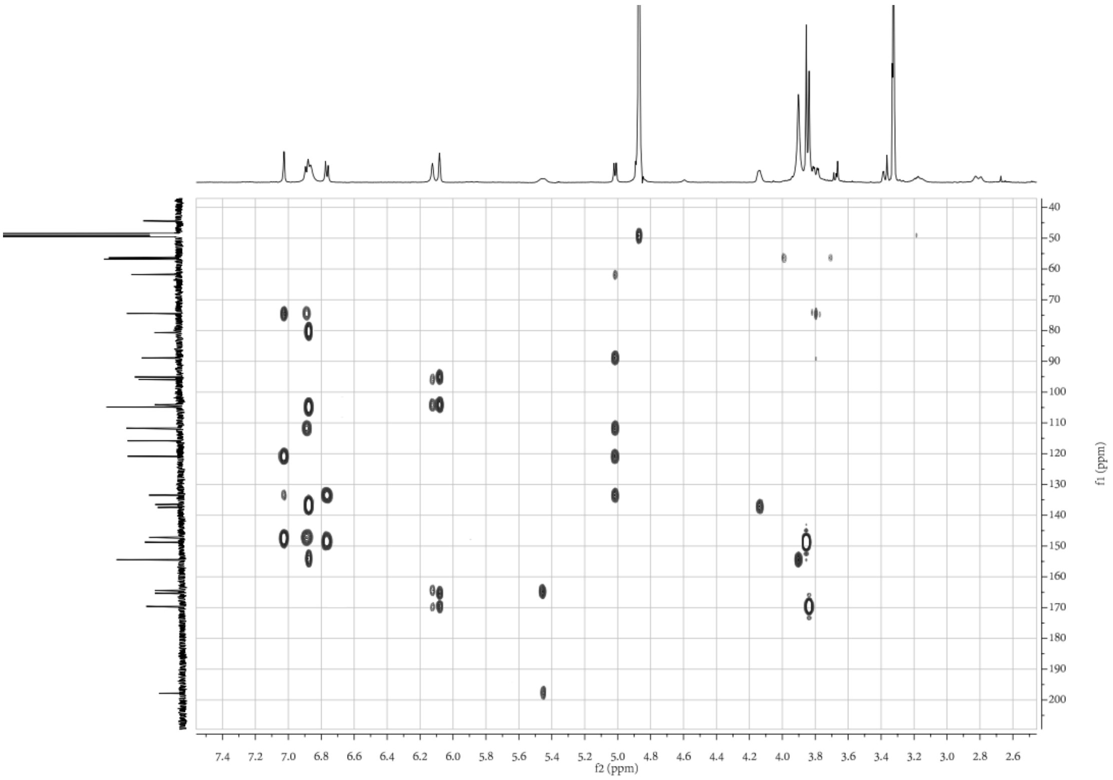

Figure S17. HMBC spectrum of **2** (CD_3OD)
